# Modern Aspects of Pernicious Anæmia.—II

**Published:** 1894-03-03

**Authors:** 


### Modern Aspects of Pernicious Anæmia.—II.

We have already given an account of the discovery and of the clinical symptoms of this disease, and now we have left the pathology, prognosis, and treatment.

The pathology of pernicious anæmia is one of the most interesting subjects in medicine. Addison frankly confessed he knew of none; all that he could foretell was that if a patient died of pernicious anæmia no cause for it would be discovered at the post-mortem; indeed, were any cause, such as cancer or phthisis, found for the anæmia, the case would not be regarded as an instance of pernicious anæmia at all. Our knowledge remained in this condition for many years, and demonstrators of morbid anatomy were accustomed to point out to the students how remarkable it was that a man should die and yet absolutely nothing could be found at the autopsy except extreme pallor of the internal organs.

During the last few years our knowledge has made immense strides, for we know now that if the diagnosis of pernicious anæmia made during life is correct, there will be found at the post-mortem examination an increase of free iron in the liver. This is, perhaps, one of the most remarkable discoveries of modern medicine, for pernicious anæmia is the only disease of which we can prophecy that nothing will be found amiss at the autopsy but a chemical abnormality in the composition of one of the chief organs of the body.

The excess of iron in the liver is easily demonstrated. There are two tests. To obtain the first a piece of the organ is dipped in a solution of sulphide of ammonium; if an excess of free iron is present the organ will turn a black colour. The other test, which is much better, is that a piece of the liver is steeped in a mixture of a solution of ferrocyanide of potassium and very weak hydrochloric acid. Slowly the surface of the piece of liver becomes of a fine blue colour owing to the development of Prussian blue. If the excess of iron is only slight, the Prussian blue is only to be seen in microscopical sections. Whether the excess is great or small, sections form beautiful microscopic objects, and show that the Prussian blue is deposited in the hepatic cells at the outer part of the lobule. Quantatitive analysis of the liver shows that this increase of free iron is very great. In health there is not enough free iron to give either of the above tests, the usual percentage of iron in the organ well freed from blood being 0·083. In pernicious anæmia the average of many cases shows that the percentage of free iron is 0·535, or seven times as much. Very often this figure is much higher, and the excess of iron may be as much as twelve or fifteen times as great as the normal amount.

With regard to other organs, there is sometimes a slight increase of iron in the spleen, but it is very slight, and in certain severe cases there is an excess of free iron in the kidneys, which very well show the Prussian blue reaction very beautifully. The excess of iron is then demonstrated to be in the renal cells and the tubes, as though it were on its way to excretion in the urine.

Now, the facts we have to harmonise are: Firstly, the anæmia; secondly, the primrose tint of the skin; thirdly, the dark colour of the urine; fourthly, the great excess of free iron in the liver and the slight excess in the kidney.

The presence of the iron in the liver naturally suggests that it arrives at that organ by the portal vein, and therefore it must come either from the spleen, the pancreas, or the gastro-intestinal tract. As the amount of free iron in the spleen is not increased, the iron in the liver can hardly come from that organ. There is no evidence of its coming from the pancreas, consequently it has been supposed, and with a good show of reason, that it comes from the gastro-intestinal tract. On this view, we suppose that in pernicious anæmia there is a great destruction of blood at the gastro-intestinal periphery of the portal system. The iron set free in this destruction being carried to the liver is there stored up, and if, as in severe cases, the amount of it is very great, a little passes into the general circulation and is excreted by the kidneys, and hence occasionally the Prussian blue reaction can be obtained in these organs.

This theory of the disease also explains the other striking facts about it, for we may well suppose that this blood destruction in the gastro-intestinal periphery of the portal system leads to the formation of pigments, one of which circulating through the skin gives it the lemon tint, and the other, being excreted in the urine, gives it the dark brown colour, due to excess of urobilin, which we have seen to be characteristic of pernicious anæmia. On this view, therefore, pernicious anæmia is a disease in which the anæmia is caused by an excessive destruction of red blood corpuscles.

The seat of this destruction is probably in the gastro-intestinal walls, and not in the interior of the stomach or the intestines, for free iron cannot be absorbed by the intestinal mucous membrane, and the excreta are not coloured black as they would be if they contained much iron.

This view of the pathology of pernicious anæmia receives considerable support from the fact that about half the patients suffering from it give a history of having had vomiting and diarrhœa quite apart from any arsenic that they may have taken. It may be that the diarrhœa in some obscure way induces the destruction of the blood, or, what is far more probable, the vomiting, diarrhœa, and blood destruction all have some common unknown cause. Naturally it has been suggested that a micro-organism is the cause of these phenomena, but of this there is at present no proof.

The prognosis of pernicious anæmia is very grave. Only two or three cases of genuine recovery have been recorded. The usual course of events is for the patient to get better for a time under treatment, then to relapse again, to again improve and again relapse. Commonly the second, and almost always the third relapse is fatal. Many authors being unaware of this tendency to relapse, have recorded cases which they believe to be instances of recovery from the disease, but no patient should be considered to have recovered unless a period of decided improvement lasts at least as long as a year.

The treatment of the disease is simple. All the patients who have improved have taken arsenic in considerable doses. Rest in bed, if the patient be weak, is of great importance. The arsenic should be continued for some months after the patient is well enough to get up. Intestinal antiseptics, such as β napthol, have been tried, but without success.

